# An intrinsic mechanism controls reactivation of neural stem cells by spindle matrix proteins

**DOI:** 10.1038/s41467-017-00172-9

**Published:** 2017-07-25

**Authors:** Song Li, Chwee Tat Koe, Su Ting Tay, Angie Lay Keng Tan, Shenli Zhang, Yingjie Zhang, Patrick Tan, Wing-Kin Sung, Hongyan Wang

**Affiliations:** 1 Neuroscience & Behavioural Disorders Program, Duke-NUS Medical School, 8 College Road, Singapore, 169857 Singapore; 2 Cancer & Stem Cell Biology Program, Duke-NUS Medical School, 8 College Road, Singapore, 169857 Singapore; 3 NUS Graduate School for Integrative Sciences and Engineering, National University of Singapore, 28 Medical Drive, Singapore, 117456 Singapore; 4 Cellular and Molecular Research, National Cancer Centre, Singapore, 169610 Singapore; 5 Cancer Science Institute of Singapore, National University of Singapore, Singapore, 119074 Singapore; 6 Genome Institute of Singapore, 60 Biopolis Street, Genome 02-01, Singapore, 138672 Singapore; 7 Department of Computer Science, National University of Singapore, Singapore, 117417 Singapore; 8 Department of Physiology, Yong Loo Lin School of Medicine, National University of Singapore, Singapore, 117597 Singapore

## Abstract

The switch between quiescence and proliferation is central for neurogenesis and its alteration is linked to neurodevelopmental disorders such as microcephaly. However, intrinsic mechanisms that reactivate *Drosophila* larval neural stem cells (NSCs) to exit from quiescence are not well established. Here we show that the spindle matrix complex containing Chromator (Chro) functions as a key intrinsic regulator of NSC reactivation downstream of extrinsic insulin/insulin-like growth factor signalling. Chro also prevents NSCs from re-entering quiescence at later stages. NSC-specific in vivo profiling has identified many downstream targets of Chro, including a temporal transcription factor Grainy head (Grh) and a neural stem cell quiescence-inducing factor Prospero (Pros). We show that spindle matrix proteins promote the expression of Grh and repress that of Pros in NSCs to govern their reactivation. Our data demonstrate that nuclear Chro critically regulates gene expression in NSCs at the transition from quiescence to proliferation.

## Abstract (web summary)

The spindle matrix proteins, including Chro, are known to regulate mitotic spindle assembly in the cytoplasm. Here the authors show that in *Drosophila* larval brain, Chro promotes neural stem cell (NSC) reactivation and prevents activated NSCs from entering quiescence, and that Chro carries out such a role by regulating the expression of key transcription factors in the nucleus.

## Introduction

The balance between neural stem cell (NSC) proliferation and quiescence is essential for neurogenesis. Its alteration is linked to neurodevelopmental disorders such as microcephaly. In the mammalian adult brain, the majority of NSCs are in a mitotic inactive, quiescent state^1^. However, they can exit quiescence and resume proliferation in response to extrinsic stimuli^2^. Understanding mechanisms controlling the quiescence and reactivation of NSCs is essential to decipher stem cell behaviours in the developing brain.

*Drosophila* larval brains NSCs, neuroblasts, also undergo the reversible transition between quiescence and reactivation. At the end of embryogenesis, NSCs enter into quiescence, depending on the Hox proteins, the temporal transcription factors and a homoeodomain transcription factor Prospero (Pros)^3–5^. NSCs are quiescent for ~24 h between embryonic and larval stages^6–10^ and are reactivated in early larval stages in response to feeding^6, 7^. Dietary amino acids are sensed by the fat body, a functional equivalent vertebrate liver and adipose tissue^11, 12^. The fat body triggers the production of insulin-like peptides by blood-brain barrier glial cells, activating the insulin/insulin-like growth factor (IGF) pathway in underlying NSCs and stimulating their reactivation^13–15^. The role of mammalian IGF-1 in NSC division^16^ and the association of human IGF1R mutations with microcephaly^17^ suggest a conserved mechanism underlying NSC proliferation. However, intrinsic mechanisms that reactivate *Drosophila* NSCs are not well established.

A unique group of mitotic regulators, termed spindle matrix proteins, functions in spindle assembly in both *Drosophila* and mammalian cells^18, 19^. In *Drosophila*, spindle matrix proteins contain at least four nuclear proteins—Chromator/Chriz (Chro), Megator (Mtor), Skeletor and enhanced adult sensory threshold (East)^20–22^. These spindle matrix proteins have intriguing cell cycle-dependent subcellular localization: they are localized to nucleus during interphase, and translocate to the mitotic spindle during mitosis^23^. While their roles during spindle assembly are well established, their nuclear function remains elusive.

Here we show that the spindle matrix complex, including Chro, Mtor and EAST, has a novel nuclear function in reactivation of *Drosophila* NSCs. Upon depletion of *chro*, *Mtor* or *east*, NSCs exhibit cellular extensions, a hallmark for quiescent NSCs, with a failure of 5-ethynyl-2′-deoxyuridine (EdU) incorporation, indicating that these NSCs were in a quiescent state. We further demonstrate that Chro is not only critical for NSC reactivation, but also essential for preventing re-entry into quiescence. Our genetic experiments show that Chro functions downstream of extrinsic insulin/IGF pathway. NSC-specific in vivo profiling has identified many potential targets of Chro including a temporal transcription factor Grainy head (grh) and a NSC quiescence-inducing factor Pros. We show that Chro activates *grh* expression while represses *pros* in NSCs at the transition of quiescence and proliferation. Our study demonstrates a critical cell-intrinsic mechanism by which Chro functions as a critical nuclear factor to control gene expression during NSC reactivation.

## Results

### Chro functions intrinsically to reactivate NSCs

**Fig. 1:**
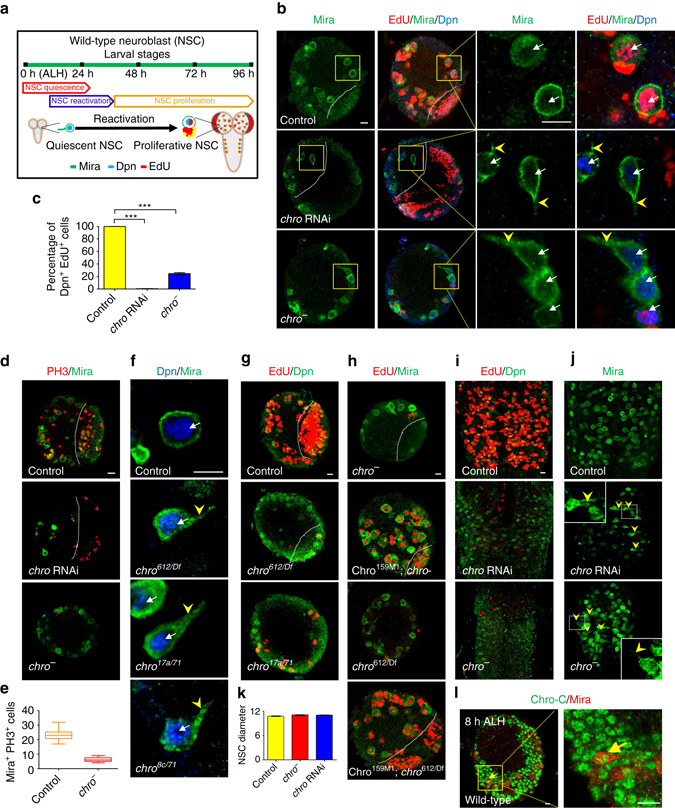
Loss of *chro* function resulted in NSCs quiescence in larval brains. **a** Schematic illustration of timing of reactivation in wild-type larval brain NSCs. **b** Third-instar larval brains in control, *chro* RNAi knockdown and *chro* ^*−*^ NSCs were labelled with Mira, Dpn and EdU. The *right* panels are enlarged views of the *yellow boxes* in the *left* panels. *chro* ^*−*^ is a trans-heterozygous mutant between *chro* ^*71*^, an N-terminal 71 amino acids truncation, and *chro* ^*612*^, a truncated Chro retaining its N-terminal 612 amino acids. **c** Quantification of percentages of Dpn^+^ EdU^+^ cells in various genotypes. **d** PH3 and Mira were labelled in control, *chro* RNAi knockdown and *chro* ^*−*^ mutant. **e** Quantification of numbers of PH3^+^ Mira^+^ cells in control (23 ± 4, *n* = 22) and *chro* ^*−*^ (6 ± 2, *n* = 21) larval brains. **f** Mira and Dpn were labelled in control, *chro* ^*612*^/Df(3L)ED231(*chro* ^*612/Df*^), *chro* ^*17a/71*^ and *chro* ^*8c/71*^ NSCs. **g** Dpn and EdU were labelled for control, *chro* ^*612/Df*^ and *chro* ^*17a/71*^ larval brains. **h** BAC clone CH322-159M1 containing genomic DNA encompassing wild-type *chro* and its promoter region (Chro^159M1^) was introduced into *chro* ^*−*^ or *chro* ^*612/Df*^ mutant background. Dpn and EdU were labelled in *chro* ^*−*^, Chro^159M1^; *chro* ^*−*^, *chro* ^*612/Df*^ and Chro^159M1^; *chro* ^*612/Df*^ larval brains. **i** Dpn and EdU were labelled in the VNC of control, *chro* RNAi knockdown and *chro* ^*−*^ mutant. **j** Control, *chro* RNAi and *chro* ^*−*^ mutant VNCs were labelled with Mira. Insets are enlarged view of the *white dotted boxes*. **k** Quantification of the diameter of third-instar larval NSCs in control, *chro* RNAi and *chro* ^*−*^ mutant. NSC diameter: control, 10.7 ± 0.1 µm; *n* = 102; *chro* RNAi, 11.0 ± 0.1 µm; *n* = 101 and *chro* ^*−*^ mutant, 11.0 ± 0.1 µm; *n* = 103. **l** Mira and Chro were stained in wild-type larval brains at 8 h ALH. The *right* panel is enlarged view of the *yellow box* in the *left* panel. *Yellow arrow* indicates wild-type quiescent NSC. All error bars indicate ± SD. in **c**, **e** and **k**. *** indicates *P* < 0.001 in **c** by Student’s *t*-test. The central brain is to the *left* of the *white dotted line* in **b**, **d**, **g**, **h**. *Arrows* indicated NSCs and *arrowheads* indicated the cellular extension of NSCs in **b**, **f** and **j**. Scale bars, 10 µm

We uncovered chromator (*chro*), a spindle matrix component, as a novel intrinsic player that reactivates NSCs, from an RNA interference (RNAi) screen (Wei X. and Wang H., unpublished data). Spindle matrix proteins are a unique group of mitotic regulators that are localized to the spindle matrix in both *Drosophila* and mammalian cells^18^. All wild-type central brain NSCs lose their cellular processes and exit quiescence by 96 hours after larval hatching (h ALH) (Fig. 1a, b; *n* = 1324). However, at 96 h ALH upon *chro* RNAi knockdown under a NSC-specific driver *insc*-Gal4, 27% (*n* = 475) of Miranda (Mira)-positive NSCs, still extended their cellular processes and only 1% (*n* = 416) of Deadpan (Dpn)-positive NSCs were incorporated with EdU (Fig. 1b, c). Twenty-six per cent (*n* = 522) of *chro* ^*71/612*^ (henceforth referred to as *chro* ^*−*^) NSCs, retained cellular extensions and only 24% (*n* = 515) of NSCs in *chro* ^*−*^ were incorporated with EdU (Fig. 1b, c). In addition, the number of mitotic cells, which are positive for Phospho-Histone H3 (PH3), was drastically reduced in *chro* ^*−*^ compared with the control (Fig. 1d, e). Likewise, hemizygous *chro* ^*612*^/Df(3L)ED231, trans-heterozygous *chro* ^*17a*^/*chro* ^*71*^ and trans-heterozygous *chro* ^*8c*^/*chro* ^*71*^ all displayed NSC reactivation defects (Fig. 1f, g). A genomic DNA encompassing wild-type *chro* gene region fully rescued the lethality, NSC quiescence and Chro expression in *chro* mutants (Fig. 1h and Supplementary Fig. 1a–c). *chro* RNAi knockdown in glial cells did not result in any delay in NSC reactivation, neither did its depletion in NSCs affect the specification, identity or death of NSCs (Supplementary Fig. 1d, e).

We analysed Chro expression in wild-type quiescent vs. active NSCs in a time-course experiment from 8 h ALH to 96 h ALH. At 8 h ALH, the expression levels of Chro in quiescent NSCs are comparable to those in active NSCs (Fig. 1l). At later stages, Chro was expressed in the nucleus of active NSCs. In addition, we examined the total protein levels of Chro by western blotting at 12 h ALH and 96 h ALH. The protein levels of Chro at both stages were similar to each other (Supplementary Fig. 2).

Similar to central brain NSCs, NSCs in the thoracic ventral nerve cord (tVNC) are reactivated in early larval stages^13^ (Fig. 1i, j). Chro is also critical for NSC reactivation in tVNC, as many tVNC Mira^+^ NSCs displayed cellular extensions at 72 h ALH and the number of EdU^+^ cycling NSCs in *chro* RNAi or *chro* ^*−*^ mutants was dramatically reduced (Fig. 1i, j). Intriguingly, the diameter of tVNC (Supplementary Fig. 1f) or central brain NSCs (Fig. 1b) from *chro* ^*−*^ mutant or *chro* RNAi is similar to that of control cycling NSCs (Fig. 1k). These observations suggest that cell growth and cell cycle control can be uncoupled when *chro* is depleted.

Mushroom body (MB) neuroblasts in *Drosophila* larval central brains are known to divide throughout larval stages in the absence of dietary amino acids and do not enter or exit from quiescence.^7^ *chro* RNAi knockdown under a MB-specific driver OK107-Gal4 resulted in the formation of shorter mitotic spindle (Supplementary Fig. 3a, b; 50%, *n* = 20), consistent with a previously known function for Chro in mitosis^20^. However, none of the MB neuroblasts (*n* = 108) displayed Mira^+^ cellular extensions and all of the MB neuroblasts were incorporated with EdU (*n* = 124) upon knockdown of *chro* under OK107-Gal4, similar to those in control MB neuroblasts (Supplementary Fig. 3c, d). These results suggest that the *chro* depletion causes a failure of NSCs to exit quiescence rather than a cell cycle arrest due to mitotic defects. Next, we directly analysed MB neuroblasts in *chro* RNAi under *insc*-Gal4 driver. At 12 h ALH, Mira^+^ MB neuroblasts from both wild type (*n* = 144) and *chro* RNAi under *insc*-Gal4 (*n* = 168), surrounded by Dachshund (Dac)-positive MB neurons, were incorporated with EdU (Supplementary Fig. 3g). Moreover, none of MB neuroblasts in control (*n* = 160) or *chro* RNAi driven by *insc*-Gal4 (*n* = 176) displayed cellular extensions (Supplementary Fig. 3g). By contrast, in the same samples, at 12 h ALH, 14% control non-MB neuroblasts (*n* = 636) were quiescent without EdU incorporation, while 45% of non-MB neuroblasts in *chro* RNAi under *insc*-Gal4 (*n* = 780) were quiescent without EdU incorporation (Supplementary Fig. 3h). These data suggest that *chro* depletion under *insc*-Gal4 causes reactivation defects of non-MB neuroblasts without affecting proliferation of MB neuroblasts. Finally, at 36 h ALH at 29 °C, similar to control (*n* = 112), MB neuroblasts from *chro* RNAi under *insc*-Gal4 (*n* = 120) continued to proliferate in the absence of dietary amino acids (Supplementary Fig. 3i). This result suggests that proliferation of MB neuroblasts is unaffected at either normal or nutrient restriction conditions upon *chro* RNAi knockdown under *insc*-Gal4. Taken together, our data explicitly support the role of Chro in regulating reactivation of NSCs.

In wild type, 97% (*n* = 96) of NSCs possess two Asterless (Asl)-positive centrioles throughout the cell cycle, due to a very short G1 in the cell cycle of *Drosophila* NSCs. However, knocking down of *chro* by RNAi by *insc*-Gal4 resulted in only 47% (Supplementary Fig. 3e; *n* = 116) of NSCs displayed two Asl-positive centrioles, suggesting that significant portion of these NSCs were in G0 or early G1. By contrast, all MB neuroblasts (Supplementary Fig. 3f; *n* = 100) with *chro* RNAi knockdown contained two Asl-positive centrioles, similar to the control MB neuroblasts. Overexpression of cyclin B (CycB), a mitotic cyclin, or cyclin E (Cyc E), a G1 cyclin, failed to rescue cellular extension phenotype observed in NSCs upon *chro* RNAi knockdown under *insc*-Gal4. Control *chro* knockdown had 26% of NSCs (*n* = 467) with cellular extensions (Supplementary Fig. 3j). Similarly, 26% (*n* = 576) and 27% (*n* = 507) of NSCs upon overexpression of CycB or CycE in *chro* RNAi displayed cellular process phenotype, respectively (Supplementary Fig. 3j). JIL-1 kinase (JIL-1), a known binding partner of Chro, which regulates spindle morphology^20^, appears to be dispensable for reactivation of NSCs (Supplementary Fig. 3k). These observations indicate that spindle/mitotic defect alone is insufficient to cause a failure in reactivation of NSCs.

The cellular extension of wild type or *chro* mutant quiescent NSCs is both actin- and microtubule-rich, as indicated by α-tubulin, disc large (Dlg) or phalloidin (Supplementary Fig. 1g, h). The thick protuberances in *chro* mutant quiescent NSCs mimic the structure of primary cilium. Acetylated-α-tubulin (Ace-Tub on lysine 40), a posttranslational modification that stabilizes microtubules observed in cilia, was strongly enriched in the cell cortex as well as the cellular protrusions in *chro* ^*−*^ mutant or *chro* RNAi NSCs (Supplementary Fig. 1i). Pericentriolar material protein γ-tubulin was strongly diminished in 95% (*n* = 155) of *chro* ^*−*^ mutant NSCs (Supplementary Fig. 1j), suggesting that centrosome maturation does not occur in these quiescent cells. Interestingly, majority of NSCs with Mira^+^ cellular extensions contained an Asl-positive centriole within or in close proximity to the cellular protrusions in *chro* ^*−*^ mutant and *chro* RNAi (Supplementary Fig. 1j). This property is similar to centriole position in primary cilia.

We performed time-course experiments to monitor the requirement of Chro for NSC reactivation. In wild type, vast majority of NSCs are incorporated with EdU and only 10% (*n* = 106) of NSCs showed cellular extensions at 24 h ALH; all NSCs lost cellular extensions and were incorporated with EdU at 48 and 72 h ALH (Supplementary Fig. 4a–d). However, significant portion of *chro* ^*−*^ NSCs still extended cellular processes and vast majority of NSCs failed to incorporate EdU from 24 to 72 h ALH (Supplementary Fig. 4a–c). Nutrition is dispensable for bulk NSC proliferation at later larval stages^7, 12, 24^. We wondered if Chro function is still required for NSC proliferation in late larval stages. When *chro* RNAi was induced at 29 °C for 2 days only after all NSCs had already reactivated, 87% (*n* = 635) of NSCs failed to incorporate with EdU and 22% of NSCs (*n* = 575) showed cellular processes (Supplementary Fig. 4e–i). Thus, *chro* depletion can result in re-entry of NSC quiescence during late larval stages. This observation suggests that Chro function is critically required for both initiation and maintenance of the proliferation status of NSCs throughout larval stages. However, overexpression of Chro did not lead to the reactivation of NSCs on amino-acid depleted condition (Supplementary Fig. 4j), suggesting that Chro is necessary but not sufficient for NSCs to exit from quiescence.

### Chro functions downstream of insulin/PI3K pathway in NSCs

**Fig. 2:**
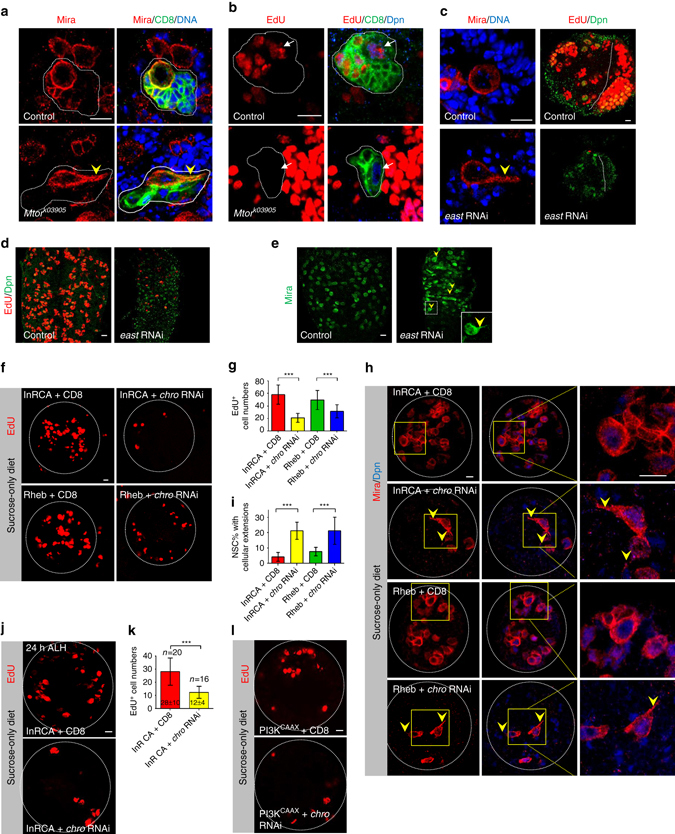
Spindle matrix proteins regulate NSC reactivation and function downstream of insulin pathway during NSC reactivation. **a** NSC MARCM clones in control and *Mtor* ^*k03905*^ were labelled with Mira, CD8 and Topro-3 (for DNA). **b** EdU, CD8 and Dpn were labelled in control and *Mtor* ^*k03905*^ NSC clones. **c** Mira and Topro-3 or Dpn and EdU were labeled in NSCs from control and *east* RNAi knockdown. **d**, **e** Dpn and EdU or Mira were labelled in the VNC of control and *east* RNAi knockdown. **f** Larval brains from overexpression of an active form of InR (OE InRCA) with UAS-CD8-GFP, overexpression of InRCA with *chro* RNAi knockdown (OE InRCA + *chro* RNAi), overexpression of RHEB (OE RHEB) with UAS-CD8-GFP, and overexpression of RHEB with *chro* RNAi knockdown (OE RHEB + *chro* RNAi) were raised on sucrose-only (amino-acid free) food for 72 h and labelled by EdU. **g** Quantification of EdU^+^ cells per brain hemisphere in various genotypes. **h** Mira and Dpn were labelled in OE InRCA with UAS-CD8-GFP, OE InRCA with *chro* RNAi, OE RHEB with UAS-CD8-GFP and OE RHEB with *chro* RNAi on sucrose-only food. The *right* panels are enlarged views of the *yellow boxes* in the *left* panels. **i** Quantification of NSCs with a cellular extension in various genotypes. **j** Larval brains from OE InRCA with UAS-CD8-GFP and OE InRCA + *chro* RNAi were raised on sucrose-only food for 24 h and labelled by EdU. **k** Quantification of EdU^+^ cells per brain hemisphere for **j**. **l** Larval brains from PI3K (PI3K^CAAX^) overexpression with UAS-CD8 or *chro* RNAi knockdown were raised on sucrose-only food for 72 h and labelled by EdU. All error bars indicate ± SD in **g**, **i** and **k**. *** indicates *P* < 0.001 in **g**, **i** and **k** by Student’s *t*-test. NSC clones were outlined by *white dotted lines* in **a** and **b**. Larval brain hemispheres were outlined by *white dotted lines* in **f**, **h**, **j**, **l**. *Arrows* indicated NSCs in **b** and *arrowheads* indicated the cellular extension of NSCs in **a**, **c**, **e** and **h**. Scale bars: 10 µm (**a**–**c**, **f**, **h**, **j**, **l**) and 20 µm (**d**, **e**)

The spindle matrix complex in *Drosophila* contains at least four nuclear proteins, Chro, Megator (Mtor), Skeletor and enhanced adult sensory threshold (East)^20–22, 25^. Next, we ascertained the role of two other spindle matrix proteins, Mtor and East, during NSC reactivation. At 96 h ALH, in the mosaic analysis with a repressible cell marker (MARCM) clones of a loss-of-function allele *Mtor* ^*k03905*^, 40% (*n* = 85) of NSCs displayed a cellular extension, in contrast to complete reactivation of wild-type NSCs (*n* = 66, Fig. 2a). Moreover, only 7% (*n* = 72) of NSCs in *Mtor* ^*k03905*^ MARCM clones were cycling with EdU labelling (Fig. 2b). Likewise, 15% (*n* = 242) of *east* RNAi NSCs retained a cellular extension at 72 h ALH and 94% (*n* = 577) of NSCs in *east* RNAi failed to incorporate EdU (Fig. 2c). Consistently, it also led to the quiescence of tVNC NSCs (Fig. 2d, e). These data indicate that the spindle matrix complex plays a critical intrinsic role in controlling NSC reactivation.

*chro* RNAi by fat-body drivers *Cg*-Gal4 and *Lsp2*-Gal4 did not cause any defects in NSC reactivation (Supplementary Fig. 5a), implying that Chro does not activate PI3K/TOR pathway in fat body. We then ascertained whether Chro functions downstream of PI3K/Akt signalling. Overexpression of an active form of insulin-like receptor (InRCA) is capable of driving NSC reactivation in the absence of dietary amino acids^13, 14^ (Fig. 2f–i); There were 58 ± 15 (*n* = 20) EdU^+^ cells per brain hemisphere at 72 h ALH, and only 4% of NSCs showed a cellular process. Overexpression of the TOR activator Ras homologue enriched in brain (RHEB) resulted in 50 ± 15 (*n* = 20) EdU^+^ cells per brain hemisphere, with 8% of NSCs displaying a cellular extension at 72 h ALH^13, 14^ (Fig. 2f–i). However, when InRCA or RHEB was overexpressed in NSCs with *chro* RNAi in amino-acid depleted food at 72 h ALH, the number of EdU^+^ cells decreased to 21 ± 7 (*n* = 21) and 31 ± 10 (*n* = 22) per brain hemisphere, respectively; 21% of NSCs displayed a Mira^+^ cellular protrusion in both genotypes (Fig. 2f–i). At 24 h ALH, the effect of InRCA overexpression was similarly suppressed by *chro* RNAi in amino-acid depleted food (Fig. 2j, k). Furthermore, *chro* RNAi significantly suppressed the effect caused by overexpression of PI3K^CAAX^, an active form of PI3K. When PI3K^CAAX^ was overexpressed in NSCs from larvae raised in amino-acid depleted food, there were 33 ± 10 EdU^+^ cells per brain lobe (*n* = 20). By contrast, when PI3K^CAAX^ was overexpressed with *chro* RNAi knockdown, the number of EdU^+^ cells decreased dramatically to 17 ± 4 per brain hemisphere (Fig. 2l; *n* = 18). Therefore, Chro regulates NSC reactivation downstream of PI3K/TOR pathway.

### In vivo profiling of Chro identified Grh as a target in NSCs

**Fig. 3:**
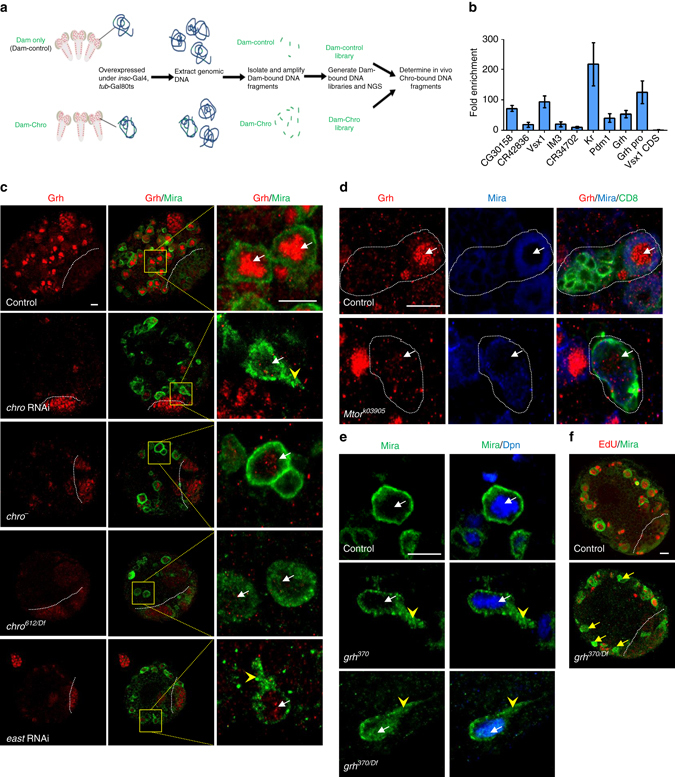
Chro promotes Grh expression during NSC reactivation. **a** A diagram of in vivo profiling of Dam-control and Dam-Chro in NSCs. **b** Quantification of fold enrichment in ChIP-qPCR assay using anti-Chro and control (anti-IgG) based on various predicted Chro-binding sites. The CDS region of *Vsx1* is used as control. Error bars indicate ± SD. **c** Control, *chro* RNAi, *chro* ^*−*^, *chro* ^*612/Df*^ and *east* RNAi third-instar larval brains were labelled with Grh and Mira. The *right* panels are enlarged views of regions in the *yellow boxes*. **d** NSC clones from control and *Mtor* ^*k03905*^ were labelled with Grh, Mira and CD8. NSC clones were outlined by *white dotted lines*. **e** Mira and Dpn were labelled in control, *grh* ^*370*^ and *grh* ^*370*^/Df(2R)PcLB (*grh* ^*370/Df*^) NSCs. NSCs with cellular extensions, control, 0%, *n* = 616; *grh* ^*370*^, 5%, *n* = 426; *grh* ^*370/Df*^, 11%, *n* = 672. **f** Larval brains from control and *grh* ^*370/Df*^ were labelled with EdU and Mira. *Yellow arrows* indicated Mira^+^ EdU^−^ NSCs in *grh* ^*370/Df*^. The central brain is to the *left* of the *white dotted line* in **c** and **f**. *White arrows* indicated NSCs in **c**, **d** and **e**, and *arrowheads* indicated the cellular extension of NSCs in **c** and **e**. Scale bars, 10 µm

To identify the targets of Chro in NSCs, we carried out targeted DNA adenine methyltransferase (Dam) identification (TaDa)-based in vivo profiling, a recently developed technique that allows cell-type-specific in vivo profiling of transcriptome and chromatin binding^26^. Dam-methylated DNA fragments were isolated and amplified from larval brains expressing Dam-Chro or Dam-control, followed by next-generation sequencing (NGS) and bioinformatics analysis (Fig. 3a). In total, we found 2269 putative Chro-binding sites with high peak scores (>100) in the whole genome (Supplementary Data 1). Five randomly selected candidate genes, namely *CG30158*, *CR42836*, *Vsx1*, *IM3* and *CR34702*, were all validated by chromatin immunoprecipitation (ChIP)-quantitative polymerase chain reaction (qPCR) analysis in S2 cells (Fig. 3b), supporting the reliability of our TaDa data. Although we cannot formally exclude the possibility that Chro binds indirectly to these targets through other chromatin-binding proteins, it was shown that N terminus of Chro interacts with histone H1^27^, suggesting that Chro directly binds to chromatin.

We found that 24% of peaks predicted were within promoter region (±1000 bp from TSS, transcriptional start sites) in our Dam-ID list, while 76% of peaks were in distal region of the genes (Supplementary Data 1). These data suggested that Chro was capable of binding to both promoter and enhancer regions of targets genes. Interestingly, Putzig (Pzg/Z4), a known chromatin insulator that interacts directly with Chro^28, 29^, is required for reactivation of NSCs. Remarkably, in the larval brains from a null allele *pzg* ^*66*^ at 96 h ALH, 23% of NSCs extended cellular protrusion (*n* = 267), and none of the NSCs were incorporated with EdU (Supplementary Fig. 5b; *n* = 714). Consistent with the above finding, knocking down of Pzg by RNAi also resulted in a failure of EdU incorporation in 73% of NSCs (*n* = 547) and cellular extension phenotype (7%, *n* = 607), compared to control NSCs with all of them incorporated with EdU (*n* = 1123) without cellular extension (Supplementary Fig. 5b; *n* = 822). These data suggest that Pzg also plays an intrinsic role in controlling NSC reactivation. Interestingly, Chro and Pzg were both associated with promoter-associated enhancers in *Drosophila* S2 cells^19^, supporting a potential role for both Chro and Pzg as nuclear architectural proteins regulating NSC reactivation.

Our Dam-Chro profiling data predicted that Chro binds to regulatory regions of temporal factors including *Kruppel* (*Kr*), *pdm1* and *grh* (Supplementary Data 1). These temporal factors control NSC proliferation or mitotic exit with obscure mechanisms^3, 30–32^. Strikingly, Chro could bind to the predicted binding sites or promoter regions of *Kr*, *pdm1* and *grh* by ChIP-qPCR (Fig. 3b). The expression of Kr or Pdm1 was unaffected in larval brains with *chro* RNAi (Supplementary Fig. 5c). At 8 h ALH, Grh is detected in the nucleus of wild-type quiescent NSCs with cellular extensions (Supplementary Fig. 5d). However, remarkably, we found that Grh expression was dramatically reduced in *chro* RNAi NSCs (98%, *n* = 920; Fig. 3c). Likewise, 61% (*n* = 526) or 74% (*n* = 1072) of interphase NSCs in *chro* ^*−*^ or *chro* ^*612*^/Df(3L)ED231 mutant showed weak or undetectable Grh expression (Fig. 3c). Furthermore, Grh levels were dramatically decreased upon East knockdown by RNAi (Fig. 3c; 66%, *n* = 236) or *Mtor* depletion (Fig. 3d; 84%, *n* = 56). These data indicate that the spindle matrix complex promotes *grh* expression in larval NSCs. Grh is reported to maintain NSC proliferation^31^ but whether its depletion results in quiescence was unknown. In a time-course experiment, we synchronized the larvae from wild type, *grh* ^*370*^ and *grh* ^*370*^/Df(2R)PcLB and examined the Mira^+^ process phenotype in the NSCs from 12 h ALH to 72 h ALH. At 12 h ALH, 15% (*n* = 224) of wild-type NSCs displayed cellular extensions and 85% (*n* = 501) of NSCs were incorporated with EdU. However, at the same time point, 28% (*n* = 315) of *grh* ^*370*^ NSCs showed cellular protrusions and only 69% (*n* = 305) of *grh* ^*370*^ NSCs were incorporated with EdU. Similarly, in *grh* ^*370*^/Df(2R)PcLB, 30% (*n* = 206) of NSCs showed cellular process and only 55% (*n* = 291) of NSCs were incorporated with EdU at 12 h ALH (Supplementary Fig. 5e). At 24 h ALH, 11% (*n* = 581) of NSCs in wild type displayed cellular extensions, while 16% (*n* = 345) and 18% (*n* = 564) of NSCs in *grh* ^*370*^ and *grh* ^*370*^/Df(2R)PcLB showed cellular process, respectively. At 48 and 72 h ALH, none of NSCs in wild type showed cellular extensions. By contrast, NSCs with cellular process phenotype were observed in *grh* ^*370*^ and *grh* ^*370*^/Df(2R)PcLB at both time points (48 h ALH: *grh* ^*370*^, 14%, *n* = 450; *grh* ^*370*^/Df(2R)PcLB, 18%, *n* = 531; 72 h ALH: *grh* ^*370*^, 14%, *n* = 419; *grh* ^*370*^/Df(2R)PcLB, 13%, *n* = 401) (Supplementary Fig. 5e). These data suggest that Grh is important at early larval stage for NSC reactivation. Consistent with these observations, at 96 h ALH in the hemizygous *grh* ^*370*^/Df(2R)PcLB mutant, 11% (*n* = 672) of NSCs showed cellular protrusions and 32% (*n* = 831) of NSC failed to incorporate EdU (Fig. 3e, f). Similarly, we also observed NSCs with cellular extensions in *grh* ^*370*^ NSCs (Fig. 3e). These observations indicate that Grh has a role in central brain NSC reactivation similar to spindle matrix proteins.

*Drosophila grh* gene spans over 40 kb in the genome and has eight transcripts, namely *grh*-RL, *grh*-RP, *grh*-RJ, *grh*-RI, *grh*-RH, *grh*-RK, *grh*-RN and *grh*-RO. In our Dam-Chro profiling data, we found a predicted peak localized at the regulatory region of *grh* (isoforms I, J, H, P or L). To explore whether Chro could regulate *grh* transcription, we performed ChIP-qPCR and identified a 423 bp regulatory region of *grh*, which was significantly enriched (Fig. 3b). These observations support the conclusion that Chro regulates the expression of Grh. Next, we ascertained whether overexpression of Grh was able to suppress the defects in the exit from quiescence in NSCs of *chro* ^*−*^ mutant brains. Grh has eight isoforms due to alternative splicing and we overexpressed Grh O, the NSC-specific isoform of Grh in *chro* ^*−*^ (*chro* ^*71/612*^) mutant background. While 26% (*n* = 755) of NSCs showed cellular processes in *chro* ^*−*^ mutant, only 15% of NSCs (*n* = 1098) displayed cellular extensions when Grh O was overexpressed in *chro* ^*−*^ mutant. Moreover, EdU incorporation was increased from 24% (*n* = 584) of NSCs to 36% (*n* = 523) of NSCs upon overexpression of Grh O (Supplementary Fig. 7d–f). The incomplete suppression was likely due to the fact that Grh is one of many targets of Chro in NSCs and that overall expression of Grh in *chro* ^*−*^ NSCs was weak, with 26% of them (*n* = 500) showed very weak Grh expression (Supplementary Fig. 7g). This result further supports our conclusion that Chro promotes the expression of Grh in NSCs during reactivation.

### Chro represses *pros* to promote NSC reactivation

**Fig. 4:**
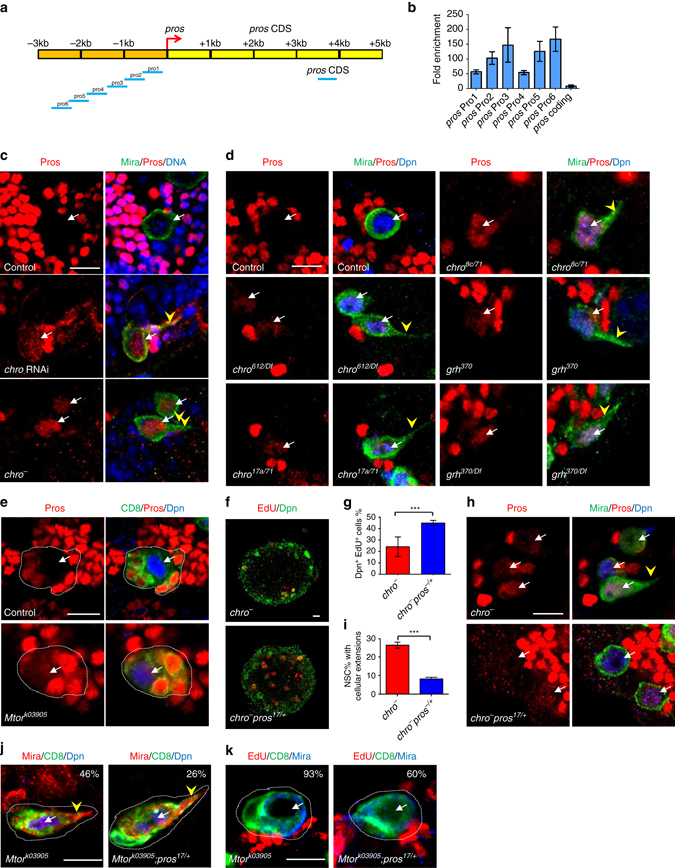
Chro prevents low-level nuclear Pros to promote and maintain NSC reactivation. **a** A schematic diagram represents *pros* regulatory region upstream of translation start site and *pros* coding sequence region. Primers designed in *pros* regulatory region (pro1-pro6) and *pros* CDS region were indicated in *blue bars*. **b** Quantification of fold enrichment in ChIP-qPCR assay using anti-Chro-C and control for different *pros* gene regions. The CDS region of *pros* is used as control. **c** Third-instar larval NSCs from control, *chro* RNAi knockdown and *chro* ^*−*^ were labelled with Mira, Pros and ToPro-3. Nuclear Pros in NSCs: control, 0%, *n* = 786; *chro* RNAi, 74%, *n* = 385; *chro* ^*−*^, 75%, *n* = 491. **d** Pros, Mira and Dpn were labelled in the NSCs of various genotypes. Nuclear Pros in NSCs: control, 0%, *n* = 627; *chro* ^*612*^/Df(3L)ED231, 71%, *n* = 984; *chro* ^*17a/71*^, 80%, *n* = 829; *chro* ^*8c/71*^, 73%, *n* = 405; *grh* ^*370*^, 50%, *n* = 522; *grh* ^*370/Df*^, 58%, *n* = 861. **e** NSC clones from control and *Mtor* ^*k03905*^ were labelled with Pros, Dpn and CD8. **f** Larval brains in *chro* ^*−*^ and *chro* ^*−*^ *pros* ^*17/+*^ were labelled by EdU and Dpn. **g** Quantification of percentages of Dpn^+^ EdU^+^ cells in *chro* ^*−*^ (24 ± 2%) and *chro* ^*−*^ *pros* ^*17/+*^ (44 ± 2%) larval brains. **h** Pros, Mira and Dpn were labelled in the NSCs in *chro* ^*−*^ and *chro* ^*−*^ *pros* ^*17/+*^. Nuclear Pros in NSCs: *chro* ^*−*^, 75%, *n* = 858; *chro* ^*−*^ *pros* ^*17/+*^, 57%, *n* = 1240. **i** Quantification of percentages of NSCs with cellular extensions in *chro* ^*−*^ (27 ± 2%) and *chro* ^*−*^ *pros* ^*17/+*^ (10 ± 1%) larval brains. **j**, **k** NSC clones from *Mtor* ^*k03905*^ or *Mtor* ^*k03905*^ *; pros* ^*17/+*^ were labelled with Dpn, Mira and CD8 or EdU, Mira and CD8. NSCs with cellular extensions: *Mtor* ^*k03905*^, 46%, *n* = 106; *Mtor* ^*k03905*^ *; pros* ^*17/+*^, 26%, *n* = 95. EdU^−^ Mira^+^ NSC clones: *Mtor* ^*k03905*^ 93%, *n* = 102; *Mtor* ^*k03905*^ *; pros* ^*17/+*^, 60%, *n* = 65. All error bars indicate ± SD in **b**, **g** and **i**. *** indicates *P* < 0.001 in **g**, **i** by Student’s *t*-test. NSC clones were outlined by *white dotted lines* in **e**, **j** and **k**. *White arrows* indicated NSCs and *arrowheads* indicated the cellular extension of NSCs. Scale bars, 10 µm

The differentiation factor Pros was detected in the nucleus of NSCs when they enter quiescence and transient expression of Pros in nucleus at low level can induce NSC quiescence^5^. Intriguingly, our Dam-Chro profiling data also predicted a Chro-binding site in *pros* regulatory region that was far from its TSS (Supplementary Data 1). To assess whether Chro could also bind to the promoter region of *pros* gene, we performed ChIP-qPCR assay. Strikingly, a 2.5 kb regulatory region near to the TSS of *pros* was dramatically enriched, compared to the control (Fig. 4a, b), suggesting that Chro binds to the promoter region of *pros*.

We wondered whether Chro suppresses premature nuclear Pros in proliferating NSCs. In wild-type larval brains, nuclear Pros was never observed in NSCs from 48 to 96 h ALH (Fig. 4c and Supplementary Fig. 6a). Remarkably, from 48 to 96 h ALH, majority of *chro* ^−^ NSCs displayed nuclear Pros localization (Fig. 4c and Supplementary Fig. 6a). Similarly, weak nuclear Pros was observed in vast majority of NSCs in *chro* RNAi, *chro* ^*612*^/Df(3L)ED231, *chro* ^*17a/71*^ and *chro* ^*8c/71*^ mutants (Fig. 4c, d). Ectopic Pros expression in NSCs for extended period of time results in premature differentiation into ganglion mother cells (GMCs) and depletion of larval NSCs^33^. However, NSCs from *chro* ^−^ or *chro* RNAi did not undergo premature differentiation, because these NSCs expressed Klumpfuss (Supplementary Fig. 6b), a transcription factor that distinguishes NSCs from GMCs^34, 35^. Importantly, NSCs were not depleted in *chro* ^−^ mutants and *chro* ^−^ NSCs were absent for a neuronal marker embryonic lethal abnormal vision (Elav) (Supplementary Fig. 6c, d). Furthermore, 91% (*n* = 773) of NSCs in *east* RNAi and 30% (*n* = 61) of *Mtor* MARCM clones displayed nuclear localization of Pros in NSCs (Fig. 4e and Supplementary Fig.  6e). In the tVNC, Grh maintains mitotic activity of NSCs by inhibiting premature nuclear Pros^30^. Similarly, central brain NSCs retained weak nuclear Pros in *grh* ^370^ or *grh* ^*370*^/Df(2R)PcLB (Fig. 4d). Taken together, the spindle matrix complex and Grh prevent low level of nuclear Pros in NSCs.

Next, we tested whether Chro regulated NSC reactivation through preventing expression of nuclear Pros in NSCs. To reduce *pros* levels, we introduced a heterozygous *pros* ^*17/+*^ mutation in *chro* mutant background, because a strong depletion of *pros* would result in NSC overgrowth and would interfere our analysis^36–39^. Remarkably, lack of EdU incorporation observed in *chro* ^−^ mutants was significantly suppressed by heterozygous *pros* ^*17/+*^ (Fig. 4f, g). Likewise, NSCs with cellular extensions or nuclear Pros localization phenotypes were significantly suppressed by heterozygous *pros* ^*17*^ in *chro* ^−^, *chro* ^*17a/71*^ or *chro* ^*8c/71*^ mutant (Fig. 4h, i and Supplementary Fig. 7a–c). In addition, notable suppression of NSC reactivation defects was observed in *Mtor* ^*k03905*^, *pros* ^*17/+*^ mutant (Fig. 4j, k). These observations indicate that the spindle matrix complex regulates Pros expression to maintain NSC proliferation. Previously, Mira degradation releases Pros from cell cortex and enter nucleus in GMCs^40, 41^. Our conclusion that Chro represses *pros* expression in NSCs may reflect two different mechanisms underlying *pros* expression within NSCs and GMCs, respectively. Next, we ascertained the genetic interaction among *chro*, *grh* and *pros*. We found that overexpression of Grh further rescued the *chro* depletion-induced NSC quiescence in *pros* heterozygotes. When Grh O was overexpressed in *chro* ^*−*^ *pros* ^*17/+*^ background, 6% (*n* = 1140) of NSCs displayed Mira^+^ cellular extensions and 54% (*n* = 1336) of NSCs were incorporated with EdU (Supplementary Fig. 7d–f). The NSC quiescent phenotype observed in Grh O; *chro* ^*−*^ *pros* ^*17/+*^ were much weaker compared to Grh O; *chro* ^*−*^ (NSC process: 15%; NSC EdU incorporation: 36%; Supplementary Fig. 7d–f) or *chro* ^*−*^ *pros* ^*17/+*^ (NSC process: 9%; NSC EdU incorporation: 45%; Fig. 4f–i) background. This result greatly supports our model that Chro regulates the expression of both *grh* and *pros* to govern NSC reactivation.

## Discussion

**Fig. 5:**
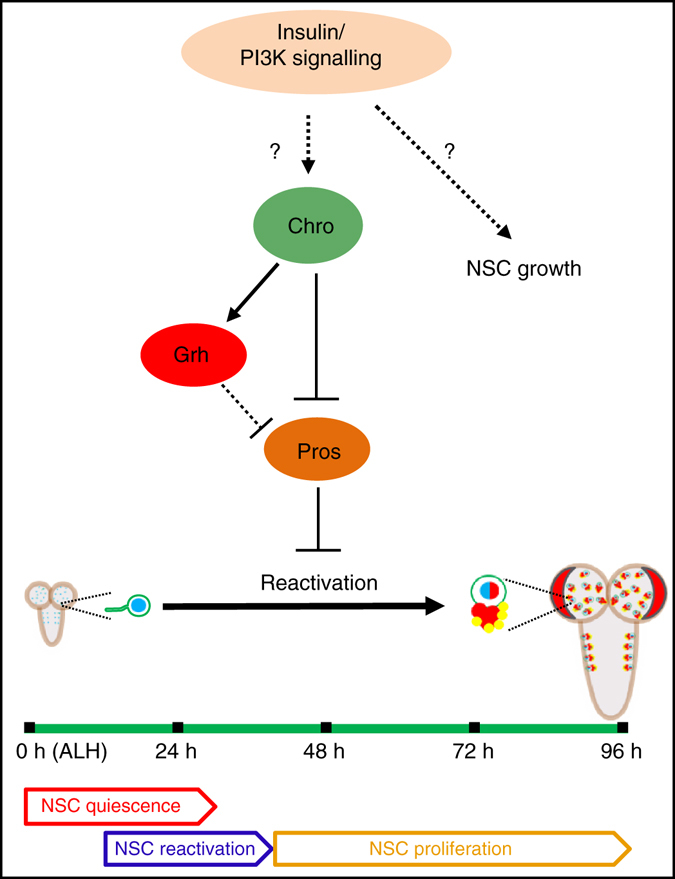
A working model. Chro functions downstream of insulin-PI3K signalling pathway and regulates the expression of Grh and Pros in NSCs during the transition from quiescence to proliferation

Here we demonstrated that the spindle matrix complex represented by Chro functions downstream of InR/PI3K/TOR signalling pathway and regulates the expression of a temporal transcription factor Grh and a quiescence-inducing transcription factor Pros to permit NSC reactivation (Fig. 5). This provides a previously missing link between extrinsic relay of signalling from blood-brain-barrier glia and subsequent activation of InR/PI3K/TOR signalling in NSCs to intrinsic transcription factors Grh and Pros that regulate reactivation or quiescence of NSCs. Recently, a role for hippo pathway in regulating NSC quiescence was reported^42, 43^. It remains to be determined whether or how Hippo pathway relates to InR/PI3K/TOR pathway and spindle matrix proteins during reactivation. Interestingly, Chro is not only essential for NSCs to exit quiescence, but also critical to prevent NSCs re-entry into quiescence at later stages when NSC growth is largely independent of nutritional requirement.

We have multiple lines of evidence to indicate that the new role of spindle matrix proteins in NSC reactivation uncovered here is distinct from their known role in mitotic spindle assembly. First, *chro* depletion in MB neuroblasts that do not enter quiescence resulted in spindle assembly defect, without causing a cell cycle arrest. Second, NSCs with *chro* depletion under *insc*-Gal4, but none of the MB neuroblasts upon *chro* depletion, appear to be in G0/G1 stage of the cell cycle. Third, overexpression of mitotic cyclin CycB or G1/S cyclin CycE was unable to rescue the quiescence phenotype of NSCs with *chro* depletion under *insc*-Gal4. Fourth, JIL-1 kinase that interacts with Chro and is required for spindle assembly in *Drosophila* S2 cells, is dispensable for NSCs to exit from quiescence. Thus, spindle matrix proteins play a novel role in regulating reactivation of NSCs independent of their known mitotic function.

The recent advances on understanding molecular signatures of mammalian quiescent stem cells, including NSCs, has largely dependent on the traditional cell isolation, sorting and profiling^44, 45^. How well these in vitro profiling data faithfully represent in vivo quiescent NSCs is difficult to assess. Our NSC-specific in vivo profiling of Dam-Chro in *Drosophila* NSCs was the first attempt of its kind towards understanding of intrinsic mechanisms of NSC reactivation and, therefore, is particularly timely. Spindle matrix proteins are speculated to play important roles in regulating genomic organization, but the mechanism remains elusive^20, 21, 23^. Our data also demonstrate, for the first time, that *Drosophila* spindle matrix protein Chro likely functions as a nuclear architectural protein in NSCs during their reactivation. First, Chro binds to thousands of sites on the genome in NSCs during their reactivation. Second, Chro is capable of both activating *grh* and repressing *pros* expression in NSCs. Third, our Dam-ID data suggested that Chro potentially binds to both promoter and enhancer regions of numerous genes in NSCs. Fourth, Pzg, a chromatin insulator protein that interacts directly with Chro, is also required for NSC reactivation. All these features of Chro resemble those of nuclear architectural proteins^46^. Taken together, Chro likely mediates long-range DNA interactions by either facilitating promoter-enhancer interaction or bringing silencers to promoters to turn off genes in NSCs during their reactivation. Our model is also supported by a recent report that Chro mediates long-range DNA interactions and is also part of an insulator complex in *Drosophila* S2 cells^47^.

Interestingly, an analogous spindle matrix with several conserved proteins is also found to be important for mitosis in mammalian cells^19^. The spindle matrix in mammalian cells is assembled by Ran GTPase, stimulating the assembly of both microtubules and Lamin B-containing matrix^48^. A recent report showed that alterations of Lamin B1 function led to cell cycle arrest and senescence in human dermal fibroblasts and keratinocytes^49^. Consistent with our finding that Chro regulates Pros in NSCs, *Drosophila* Bj1, the guanine-nucleotide exchange factor for Ran GTPase, also promotes Pros nuclear export and NSC self-renewal^50^. Ran has a well-established role in nuclear transport^51^. Interestingly, translocated promoter region (Tpr), the mammalian homologue of *Drosophila* Mtor, is a component of the nuclear pore complex and may directly regulate cell cycle genes in nucleoplasm^52^. Therefore, spindle matrix proteins may have an evolutionarily conserved intrinsic function to regulate NSC reactivation/proliferation.

## Methods

### Fly stocks and genetics

Fly stocks used in this study were: *chro* ^*8c*^ (Wasser, M); *chro* ^*17a*^ (Wasser, M); *chro* ^*71*^ (Wasser, M); *chro* ^*612*^ (Wasser, M); UAS-Chro (Wasser, M); *pros* ^*17*^ (Doe, C.Q); UAS-Grh O (Bray SJ); *grh* ^*370*^ (Bray SJ); *pzg* ^*66*^ (Nagel, A.C.); UAS-CycB (Lehner C.); UAS-CycE (Richardson H.); OK107-Gal4 (L. Luo); *Jil-1* ^*Z2*^ (K.M. Johansen). The following fly stocks were obtained from Bloomington Drosophila Stock Center (BDSC): UAS-InR.A1325D (InRCA: InR constitutively active, BDSC8440); UAS-Rheb (BDSC9689); UAS-PI3KCAAX (BDSC25908); *chro* deficiency (Df(3L)ED231; BDSC8090); *grh* deficiency (Df(2R)Pcl7B; BDSC3064); *east* RNAi (BDSC33879). *chro* RNAi (v101663) and *pzg* RNAi (v25542) were obtained from Vienna Drosophila Resource Center (VDRC). *Mtor* ^*k03905*^ was obtained from Kyoto *Drosophila* genetic resource center (DGRC 111163). To induce RNAi knockdown of various genotypes, *Drosophila* larvae were kept at 29 °C by *insc*-Gal4 unless otherwise stated. To generate transgenic fly stocks containing genomic DNA encompassing wild-type *chro* and its promoter region, a P(acman) BAC clone, CH322-159M1 was integrated into BDSC9736 (y^1^ w^1118^; PBac^7^VK00018) genome^53^.

To deprive larvae of dietary amino acids, larvae hatching within a 2-h window were transferred into amino acids-free medium (5% sucrose, 1% agar in phosphate-buffered saline (PBS)) and raised at 29 °C for 3 days before larval brains were dissected.

### Immunohistochemistry

*Drosophila* larvae were dissected in PBS, and larval brains were fixed in 3.7% formaldehyde with PBS + 0.3% Triton-100 (PBT) for 15 min. The samples were processed for immunostaining as previously described^54^. Confocal images were taken from LSM710 and brightness and contrast were adjusted by Photoshop CS5.1. Primary antibodies used in this paper were guinea pig anti-Dpn (1:1000; J. Skeath), mouse anti-Mira (1:50, F. Matsuzaki), rabbit anti-Mira (1:500, W. Chia), mouse anti-Grh (1:10, AH Brand), rat anti-CD8 (1:250, Invitrogen, Cat#: MCD0800), rabbit anti-GFP (1:500, Molecular Probes, Cat#: A21311), rat anti-Elav (1:10, DSHB, Cat#: Rat-Elav-7E8A10), mouse anti-Pros (1:10, DSHB, Cat#: Prospero (MR1A)), mouse anti-Dac (1:5, DSHB, Cat#: mAbdac2-3), mouse anti-α-tubulin (1:200, Sigma, Cat#: T6199), guinea pig anti-Asl (1:200, G Rogers), mouse anti-γ-Tub (1:200, Sigma, Cat#: T5326), mouse Acetylated-Tub (1:100, Sigma, Cat#: T7451), rat anti-Kr (1:100, EADC, Cat#: 574), rabbit anti-Pdm1 (1:500, W. Chia & X. Yang), rabbit anti-PH3 (1:200, Sigma, Cat#: 06‐570), mouse anti-Dlg (1:200, DSHB, Cat#: 4F3 anti-discs large), rabbit anti-Klumpfuss (1:200, X. Yang) and guinea pig anti-Chro-C (1:500, this study). DNA was labelled by ToPro-3 (1:5000, Invitrogen, Cat#: T3605).

### Clonal analysis

To generate NSC MARCM clones^55^, *Drosophila* larvae were heat shocked at 37 °C for 90 min at 24 h ALH. The larvae were heat shocked one more time after 10–16 h from the first heat shock and then aged for 3 days at 25 °C prior to dissection.

### EdU incorporation

*Drosophila* larvae were fed with standard food supplemented with EdU with a concentration of 0.2 mM for 4 h. Larval brains were dissected in PBS and fixed with 3.7% formaldehyde in PBT (PBS + 0.3% Triton-100) for 15 min. The brains were washed twice in PBT for 10 min, followed by 45 min blocking in 3% bovine serum albumin (BSA) in PBT. Incorporated EdU was then detected according to the Click-iT EdU protocol (Invitrogen). After EdU labelling, larval brains were rinsed twice by PBT, blocked by 3% BSA in PBT for 30 min and followed by standard immunohistochemistry.

### Quantification of cellular extensions and EdU incorporation

*Drosophila* larval brains from various genotypes were placed dorsal side up on confocal slides. The confocal z-stacks were taken from the surface to the deep layers of the larval brains (12–15 slides per z-stack with 6 µm intervals). For each genotype, at least 15 brain lobes were imaged for z-stacks and ImageJ or Zen software were used for quantifications.

### Antibody generation

The C-terminal coding sequence (encoding 345–926 amino acids) of Chro (Chro-C) was amplified by PCR and subcloned into the vector pMAL-c2X (In-Fusion HD Cloning Kit, Clontech). The expression of MBP-Chro-C was induced by isopropyl β-D-1-thiogalactopyranoside and purified by amylose resin (NEB). MBP-Chro-C was then injected into one guinea pig, and Chro-C antibodies were generated and purified by GenScript (Hong Kong). Oligos used for Chro-C cloning are:

MBP-Chro-345F (5′-AGGATTTCAGAATTCGGATCCTCCTCGCCTTCCTCG-3′) and MBP-Chro-926R (5′-TTGCCTGCAGGTCGATTACGTTGGGATGTTGAGCGT-3′).

### Western blotting

Approximately 50 brains at 96 h ALH or 100 brains at 24 h ALH from wild type were homogenized with RIPA buffer (1% NP-40; 0.1% deoxycholate; 0.1% SDS; 150 mM NaCl and 50 mM, pH 8.0, Tris-Cl) with proteases inhibitors. The protein extracts were separated by SDS-PAGE and probed with guinea pig anti-Chro-C (1:1000, this study) and mouse anti-α-tubulin (1:10,000, Sigma, Cat#: T6199). Two replica of experiments were used for quantification in ImageJ.

### TaDa in vivo profiling

To identify in vivo Chro-bound DNA fragments, full-length of Chro was cloned into pUASTattB-LT3-NDam (Brand AH) vector using In-Fusion HD Cloning Kit. UAS-Dam-Chro (pUASTattB-LT3-NDam-Chro) or UAS-Dam control (pUASTattB-LT3-NDam) was expressed under the control of *insc*-Gal4, *tub*-Gal80ts driver. Embryos were collected in a 4-h period and raised at 25 °C for 22 h. Hatched larvae were transferred into 18 °C (restrictive temperature) for 4–5 days and then shifted into 29 °C for 24 h before dissection. Genomic DNA of Dam-Chro or Dam control was extracted and amplified as previously described, except that 800 larval brains per sample were dissected^26^. Libraries were constructed from the amplified DNA samples using the illumina TruSeq DNA PCR-free library preparation kit according to the manufacturer’s protocol and sequenced on the illumina MiSeq equipment. Paired reads (2 × 250 bp) were sequenced from Dam-Chro or Dam-Control library and were aligned on *Drosophila melanogaster* (dm6) reference genome using Bowtie 2^56^. The peaks were called by MACS method^57^, and the nearest gene was annotated using HOMER^58^. Two biological replicates were performed for the profiling and analysis, and 2269 overlapping peaks (peak scores >100) from two replicates were recorded in Supplementary Data 1.

### ChIP-qPCR assay

The ChIP assay was performed using *Drosophila* S2 cells according to the manufacturer’s protocol (Millipore). To pull down the chromatin, 2 µl of guinea pig anti-Chro-C or IgG (Millipore, as a control) was used, and 1% of the precipitated DNA was used for real-time qPCR per reaction. To validate predicted Dam-Chro-binding fragments, primers for ChIP-qPCR are designed nearby the predicted region (*Pdm1, grh, IM3, CG30158, CR34702* and *Vsx1*). For those genes that have predicted Chro-binding regions far from the transcription start site, the primers are designed nearby the promoter region of the genes (*Kr, CR42836* and *pros)*. The primers used in ChIP-qPCR were listed in Supplementary Table 1.

### Statistics

Statistical analysis among different groups was performed by the Student’s *t*-test, and a value of *P* < 0.05 was considered as statistical significant. All the data are shown as mean ± SD. In this work, * indicates *P* < 0.05 and *** indicates *P* < 0.001.

### Data availability

The data sets generated during and/or analysed during the current study are available from the corresponding author on reasonable request.

## Electronic supplementary material

Supplementary Information

Supplementary Data 1
