# Erratum: An intrinsic mechanism controls reactivation of neural stem cells by spindle matrix proteins

**DOI:** 10.1038/s41467-017-01017-1

**Published:** 2017-10-31

**Authors:** Song Li, Chwee Tat Koe, Su Ting Tay, Angie Lay Keng Tan, Shenli Zhang, Yingjie Zhang, Patrick Tan, Wing-Kin Sung, Hongyan Wang

**Affiliations:** 1 Neuroscience and Behavioural Disorders Program, Duke-NUS Medical School, 8 College Road, Singapore, 169857 Singapore; 2 Cancer and Stem Cell Biology Program, Duke-NUS Medical School, 8 College Road, Singapore, 169857 Singapore; 3 NUS Graduate School for Integrative Sciences and Engineering, National University of Singapore, 28 Medical Drive, Singapore, 117456 Singapore; 4 Cellular and Molecular Research, National Cancer Centre, Singapore, 169610 Singapore; 5 Cancer Science Institute of Singapore, National University of Singapore, Singapore, 119074 Singapore; 6 Genome Institute of Singapore, 60 Biopolis Street, Genome 02-01, Singapore, 138672 Singapore; 7 Department of Computer Science, National University of Singapore, Singapore, 117417 Singapore; 8 Department of Physiology, Yong Loo Lin School of Medicine, National University of Singapore, Singapore, 117597 Singapore

*Nature Communications* **8**:122 doi: 10.1038/s41467-017-00172-9; Article published online: 25 Jul 2017

The original version of this Article contained errors in the Abstract. ‘Chro also prevents NSCs from ire-entering quiescence at later stages. NSC-specific in vivo profiling has dentified many downstream targets of Chro, including a temporal transcription factor Grainy head (Grh) and a neural stem cell quiescence-inducing factor Prospero (Pros)’ now reads ‘Chro also prevents NSCs from re-entering quiescence at later stages. NSC-specific in vivo profiling has identified many downstream targets of Chro, including a temporal transcription factor Grainy head (Grh) and a neural stem cell quiescence-inducing factor Prospero (Pros).’ These errors have now been corrected in both the PDF and HTML versions of this Article.

