# Supplementary material for: An intrinsic mechanism controls reactivation of neural stem cells by spindle matrix proteins
Source: Nat Commun. 2017 Jul 25;8:122. doi: 10.1038/s41467-017-00172-9 (PMC5526931; doi:10.1038/s41467-017-00172-9)
Supplement: Supplementary file 1 — Supplementary Information [file 41467_2017_172_MOESM1_ESM.pdf]

File Name: Supplementary Information
Description: Supplementary Figures and Supplementary Table.

File Name: Supplementary Data 1
Description: Identification of putative Chro-binding sites in the whole genome within neural stem cells by cell type-specific *in vivo* profiling.

Supplementary Figure 1

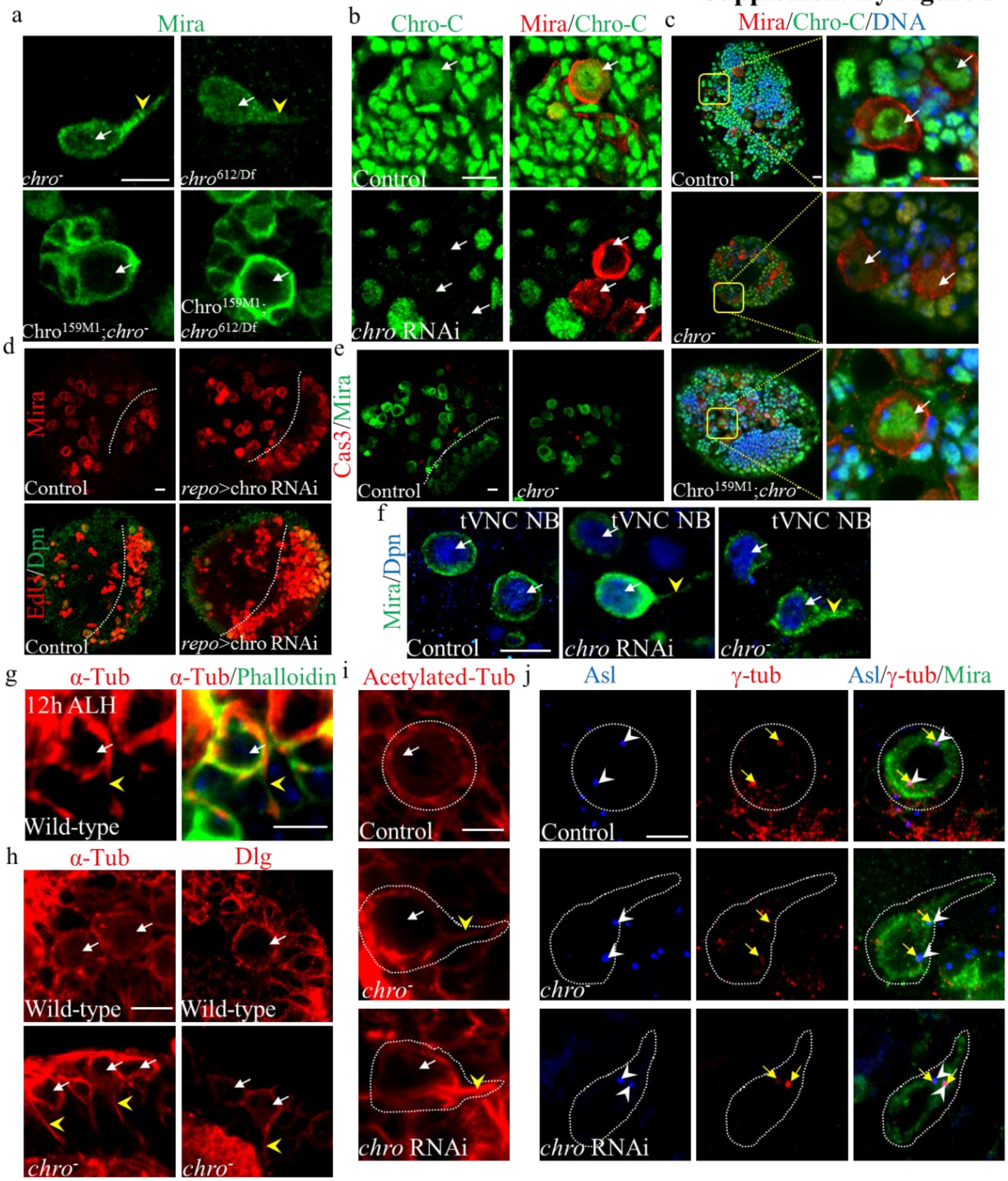

Supplementary Figure 1. Chro regulates NSC reactivation. (**a**) BAC clone CH322-159M1 containing genomic DNA encompassing wild-type *chro* and its promoter region (Chro^{159M1}) was introduced into *chro*$^{17/612}$(*chro*$^{-}$) or *chro*^{612}/Df(3L)ED231(*chro*$^{612/Df}$) mutant background. Mira was labelled in *chro*$^{-}$, Chro^{159M1}; *chro*$^{-}$, *chro*$^{612/Df}$ and Chro^{159M1}; *chro*$^{612/Df}$ neural stem cells (NSCs). (**b**) Mira and Chro-C were labelled in control and *chro* RNAi knockdown NSCs. (**c**) Mira, Chro-C

and ToPro-3 (for DNA) were labelled for control, *chro*$^-$ and Chro^{159M1}; *chro*$^-$ larval brains. The right panels are the enlarged view of the yellow boxes in left panels. (**d**) Mira or EdU and Dpn were labelled for larval brains from control and *chro* RNAi knockdown under *repo*-Gal4. (**e**) Larval brains of control and *chro*$^-$ were labeled with active caspase 3 (Cas-3) and Mira. The central brain is to the left of the white dotted line in D and E. (**f**) Dpn and Mira were labelled in VNC NSCs of control, *chro* RNAi knockdown and *chro*$^-$ mutant. (**g**) NSCs in wild-type were labelled with α-tubulin (α-Tub) and Phalloidin at 12h ALH. (**h**) Control and *chro*$^-$ mutant NSCs were labelled with α-Tub or Discs large (Dlg) at 96h ALH. (**i**) Control, *chro*$^-$ mutant and *chro* RNAi knockdown NSCs were labelled with Acetylated-tubulin (Acetylated-Tub). (**j**) Asterless (Asl), γ-tubulin (γ-tub) and Mira were labelled in NSCs from control, *chro*$^-$ mutant and *chro* RNAi knockdown. NSCs with Mira$^+$ cellular extensions containing an Asl$^+$ centriole within or in close proximity to the cellular protrusions: control, 0%, n=110; *chro*$^-$, 64%, n=72; chro RNAi, 68%, n=75. White arrowheads indicated Asl$^+$ centrioles and yellow arrows indicated γ-tub$^+$ staining. NSCs were outlined by white dotted lines in i and j. White arrows indicated NSCs in a-c and f-i, and yellow arrowheads indicated the cellular extension of NSCs in a, f-i. Scale bars, 10 µm.

Supplementary Figure 2

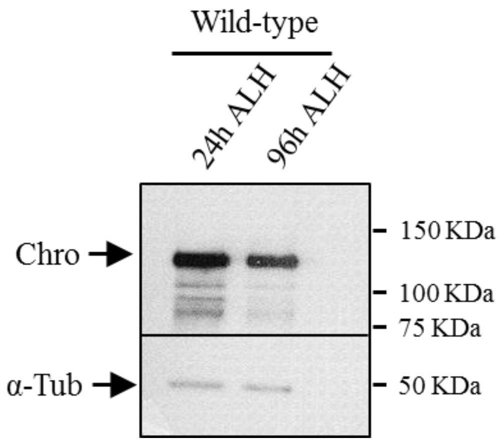

Supplementary Figure 2.Chro protein levels at 24h ALH and 96h ALH in larval brains. Protein extracts from wild-type larval brains at 24h ALH and 96 ALH were probed by anti-Chro-C and anti-α-tubulin antibodies in western blotting. After normalization and quantification in image J, the Chro levels in wild-type larval brains at 24h ALH were 1.1 times of those at 96h ALH.

Supplementary Figure 3

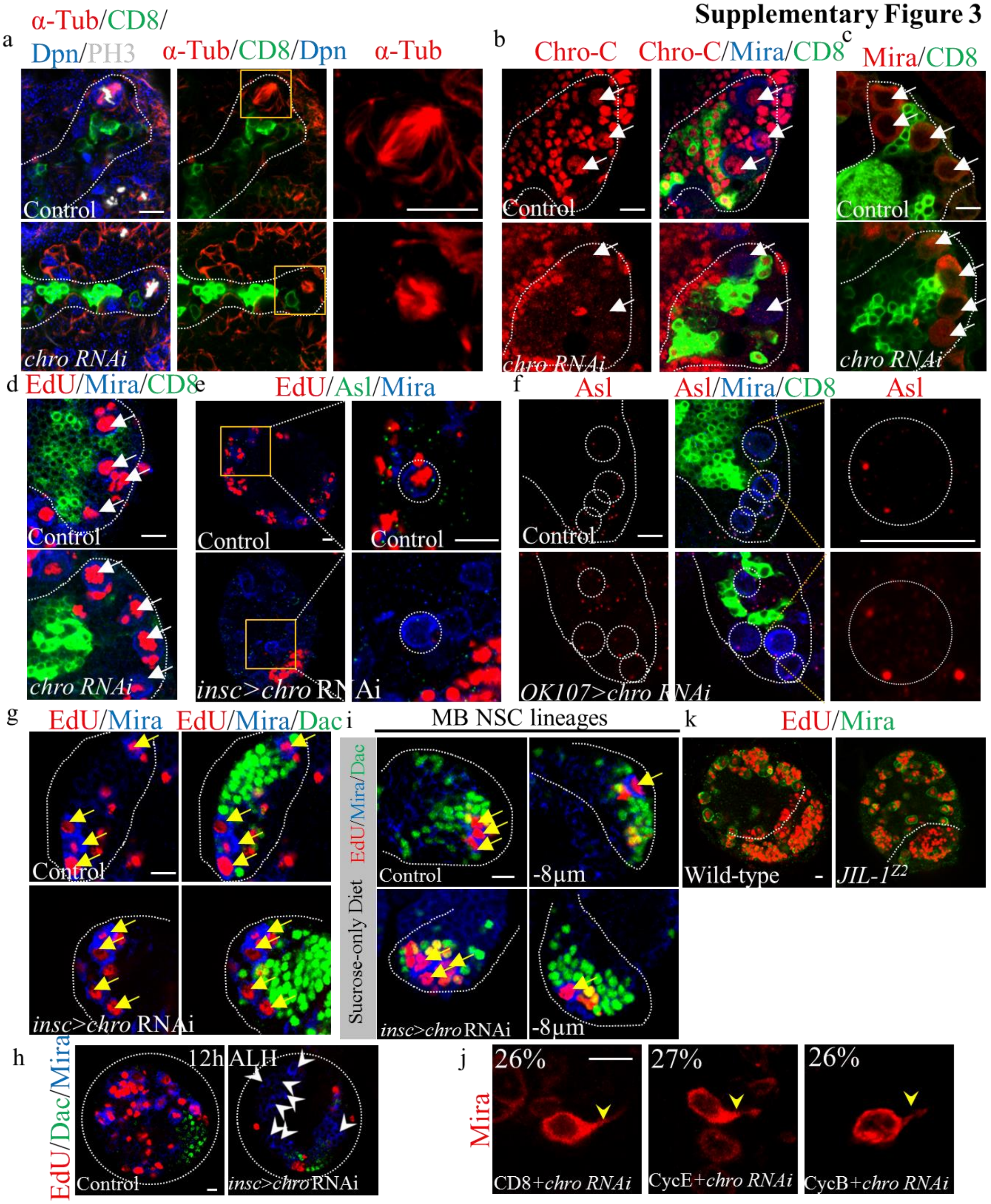

Supplementary Figure 3. Chro depletion does not obviously affect NSC proliferation in Mushroom body neuroblasts. (**a**) Dpn, α-tubulin, Phospho-Histone H3 (PH3) and CD8 were stained in control (additional UAS-CD8-GFP) and *chro* RNAi driven by Mushroom body (MB) specific driver OK107-Gal4 with UAS-CD8-GFP. The right panels are enlarged view of the yellow boxes in middle panels. (**b**) Chro-C, Mira and CD8 were labelled in control (additional

UAS-CD8-GFP) and *chro* RNAi driven by OK107-Gal4 with UAS-CD8-GFP. (**c-d**) Mira and CD8 (**c**) or Mira, EdU and CD8 (**d**) were labelled in control (additional UAS-CD8-GFP) and *chro* RNAi driven by OK107-Gal4 with UAS-CD8-GFP. (**e**) EdU, Asl and Mira were labelled in control (additional UAS-CD8-GFP) and *chro* RNAi NSCs driven by *insc*-Gal4. The middle and right panels are enlarged view of the yellow boxes in left panels. NSCs were outlined by white dotted lines. (**f**) MB NSCs from control (additional UAS-CD8-GFP) and *chro* RNAi driven by OK107-Gal4 with UAS-CD8-GFP were stained with Asl, Mira and CD8. MB neuroblast lineages were outlined by white dotted lines. The right panels are enlarged view of the MB neuroblasts in middle panels. (**g-h**) NB NSC lineages at 12h ALH were labelled by EdU, Mira and Dachshund (Dac) in control (UAS-CD8-GFP) and *chro* RNAi driven by *insc*-Gal4. Yellow arrows in **g** pointed MB NSCs in the lineage. White arrow heads in **h** indicate non-MB NSCs which failed to be incorporated with EdU. (**i**) Larval brains from control (UAS-CD8-GFP) and *chro* RNAi driven by *insc*-Gal4 were raised on sucrose-only (amino acid-free) food supplemented with EdU for 36h at 29 °C. MB NSCs lineages were labelled with EdU, Mira and Dac. Yellow arrows pointed MB NSCs in the lineage. (**j**) NSCs from *chro* RNAi with UAS-CD8, *chro* RNAi with UAS-CycE and *chro* RNAi with CycB were driven by *insc*-Gal4 and labelled by Mira. (**k**) Larval brains from wild-type and *Jil-1*^{Z2} mutant were labelled with EdU and Mira. MB neuroblast lineages were outlined by white dotted lines in a-d, f-g and i. MB neuroblasts were pointed by white arrows in b-d. Scale bars: 10 µm.

Supplementary Figure 4

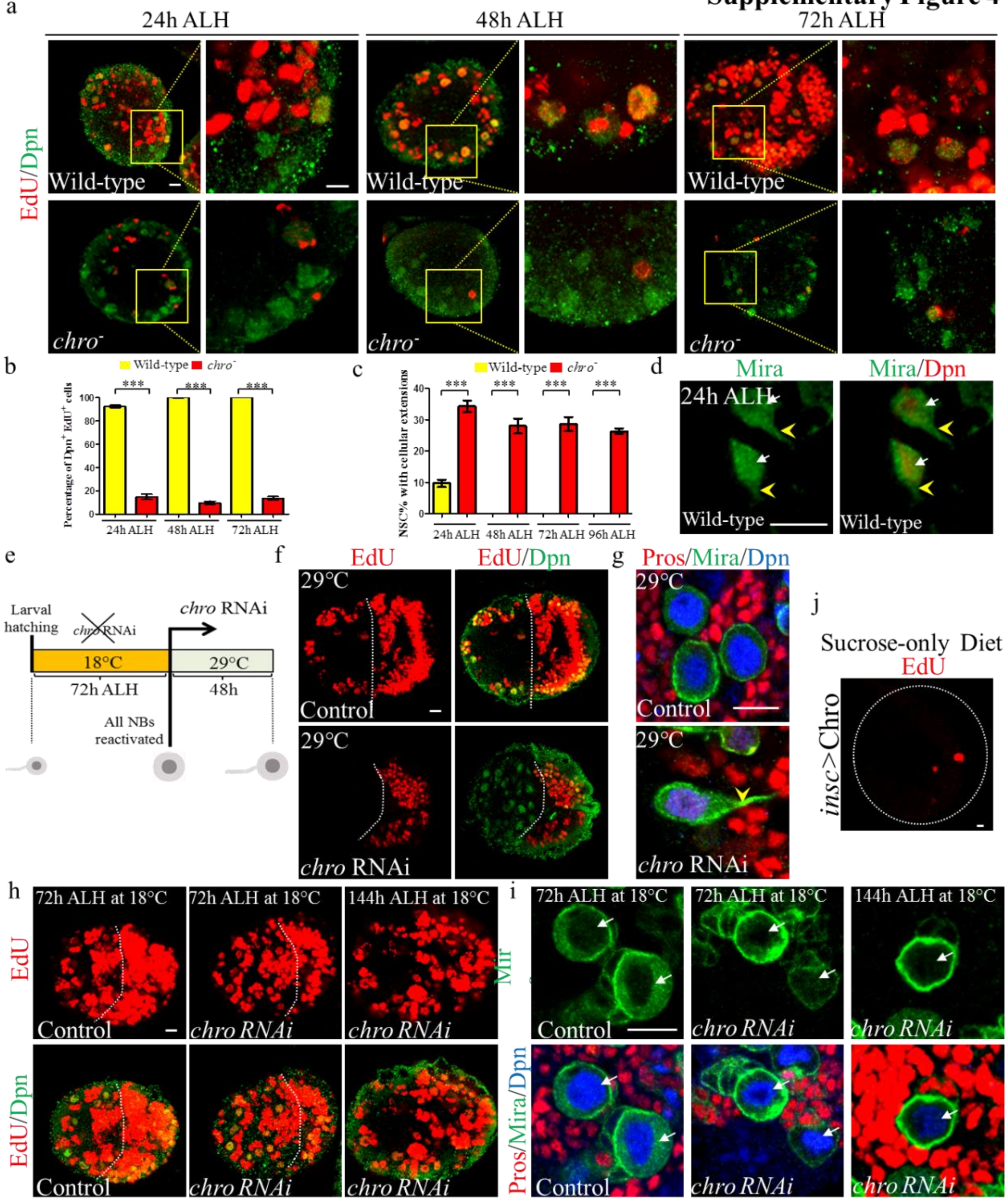

Supplementary Figure 4. Chro is important for the initiation and maintenance of NSC reactivation. (**a**) EdU and Dpn were labelled in wild-type and *chro*⁻ mutant larval brains at 24h ALH, 48h ALH or 72h ALH. The right panels are the enlarged view of the yellow boxes in left panels. (**b**) Quantification of Dpn^+ EdU^+ cells in wild-type and $chro^-$ mutant central brains. Wild-type, 24h ALH, 92%, n=992; $chro^-$, 24h ALH, 15%, n=671; wild-type, 48h ALH, 100%, n=1353;

chro$^-$, 48h ALH, 10%, n=390; wild-type, 72h ALH, 100%, n=1152; *chro*$^-$, 72h ALH, 14%, n=408. (**c**) Quantification of NSCs with cellular extensions in control and *chro*$^-$ mutant. Wild-type, 24h ALH, 10%, n=134; *chro*$^-$, 24h ALH, 35%, n=71; wild-type, 48h ALH, 0%, n=382; *chro*$^-$, 48h ALH, 28%, n=128; wild-type, 72h ALH, 0%, n=480; *chro*$^-$, 72h ALH, 29%, n=170; wild-type, 96h ALH, 0%, n=249; *chro*$^-$, 96h ALH, 26%, n=197. All error bars indicate ± s.d. in b and c. *** indicates $p<0.001$ in b, c by student's t-test. (**d**) Dpn and Mira were labelled for NSCs in wild-type at 24h ALH. (**e**) A schematic diagram showing the induction of control or *chro* RNAi knockdown at a later larval stage by *insc*-Gal4 under the control of a temperature-sensitive *tub*-Gal80^{ts}. (**f-g**) Hatched larvae from control or *chro* RNAi knockdown were raised at 18°C for 3 days and transferred to 29°C for 2 days. (**f**) EdU and Dpn or (**g**) Mira, Pros and Dpn were labelled in control and *chro* RNAi larval brains after 2 days at 29°C. (**h-i**) Control and *chro* RNAi were kept at 18°C. (**h**) EdU and Dpn or (**i**) Mira, Pros and Dpn were labelled in control and *chro* RNAi larval brains at 72h ALH or 144 ALH. (**j**) Larval brains from *insc*>Chro were raised on sucrose-only (amino acid-free) food for 72h and labelled with EdU. Larval brain lobe was outlined by white dotted lines. The central brain is to the left of the white dotted line in f and h. Arrows indicated NSCs in d, i and arrowheads indicated the cellular extension of NSCs in d, g. Scale bars, 10 µm.

Supplementary Figure 5

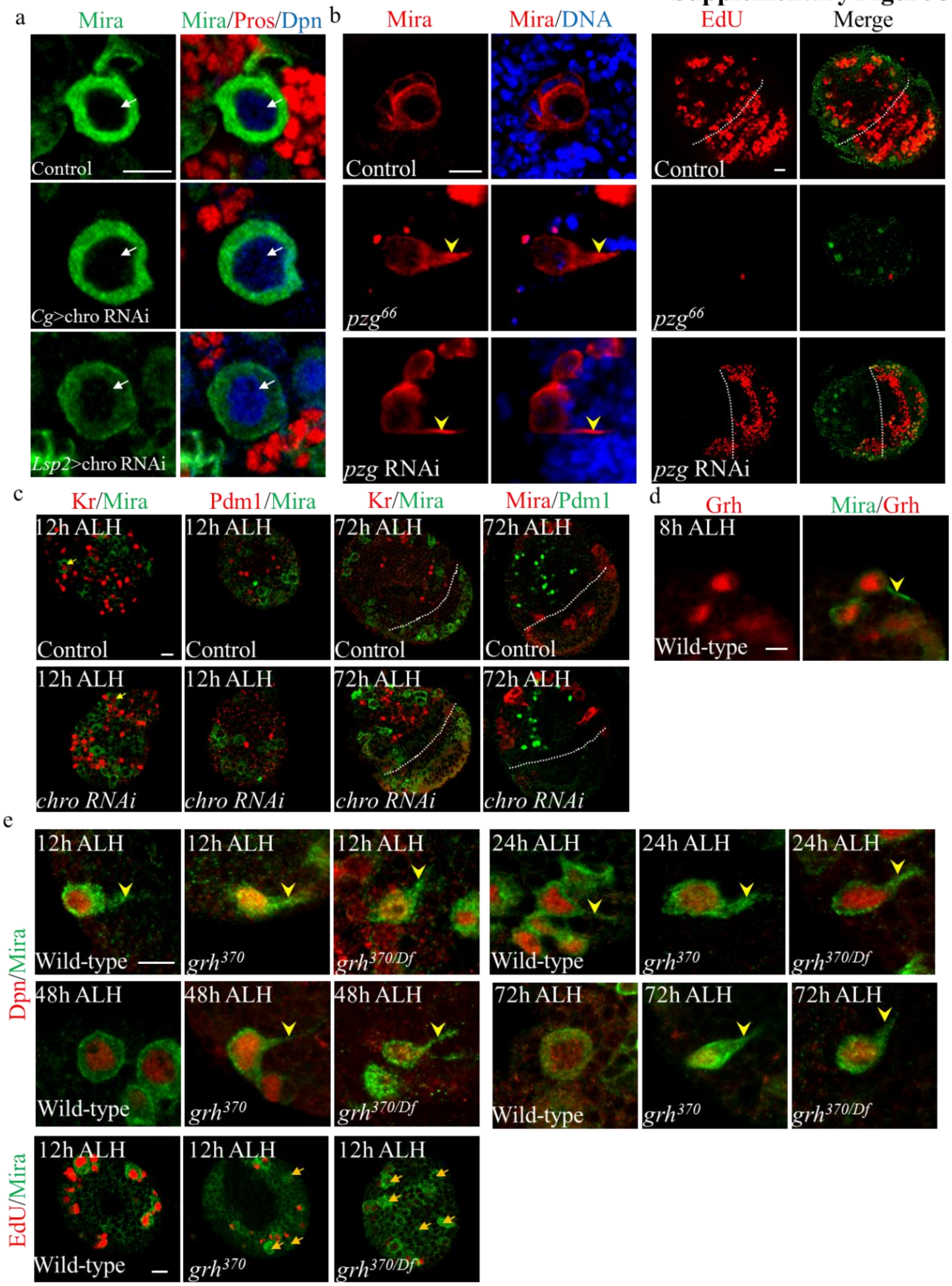

Supplementary Figure 5. Chromatin insulator protein Pzg regulates the exit of NSCs from quiescence. (**a**) NSCs (white arrows) from control, *Cg>chro* RNAi and *Lsp2> chro* RNAi were

labelled with Dpn, Mira and Pros. (**b**) Mira and DNA or EdU and Dpn were labelled for NSCs from control, *pzg*^{66} homozygous mutant and *pzg* RNAi driven by *insc*-Gal4. (**c**) Mira and Kruppel (Kr) or Pdm1 were labelled for control and *chro* RNAi knockdown larval brains for 12h ALH or 72h ALH at 29°C. Yellow arrows indicated Mira^{+} NSCs with weak Kr expression in control and *chro* RNAi at 12h ALH. At 72h ALH, Kr was undetectable in control or *chro* RNAi. Pdm1 was undetectable in control or *chro* RNAi NSC at 12h ALH and 72h ALH. The central brain is to the left of the white dotted line in b-c. (**d**) Wild-type NSCs at 8h ALH were labelled with Grainyhead (Grh) and Mira. (**e**) Mira and Dpn or EdU and Mira were labelled in wild-type, *grh*^{370} and *grh*^{370}/Df(2R)PcLB (*grh*$^{370/Df}$) NSCs at 12h, 24h, 48h and 72h ALH. NSCs without EdU incorporation were indicated by yellow arrows in e. Yellow arrowheads indicated the cellular extension of NSCs in b, d and e. Scale bars, 10 µm.

Supplementary Figure 6

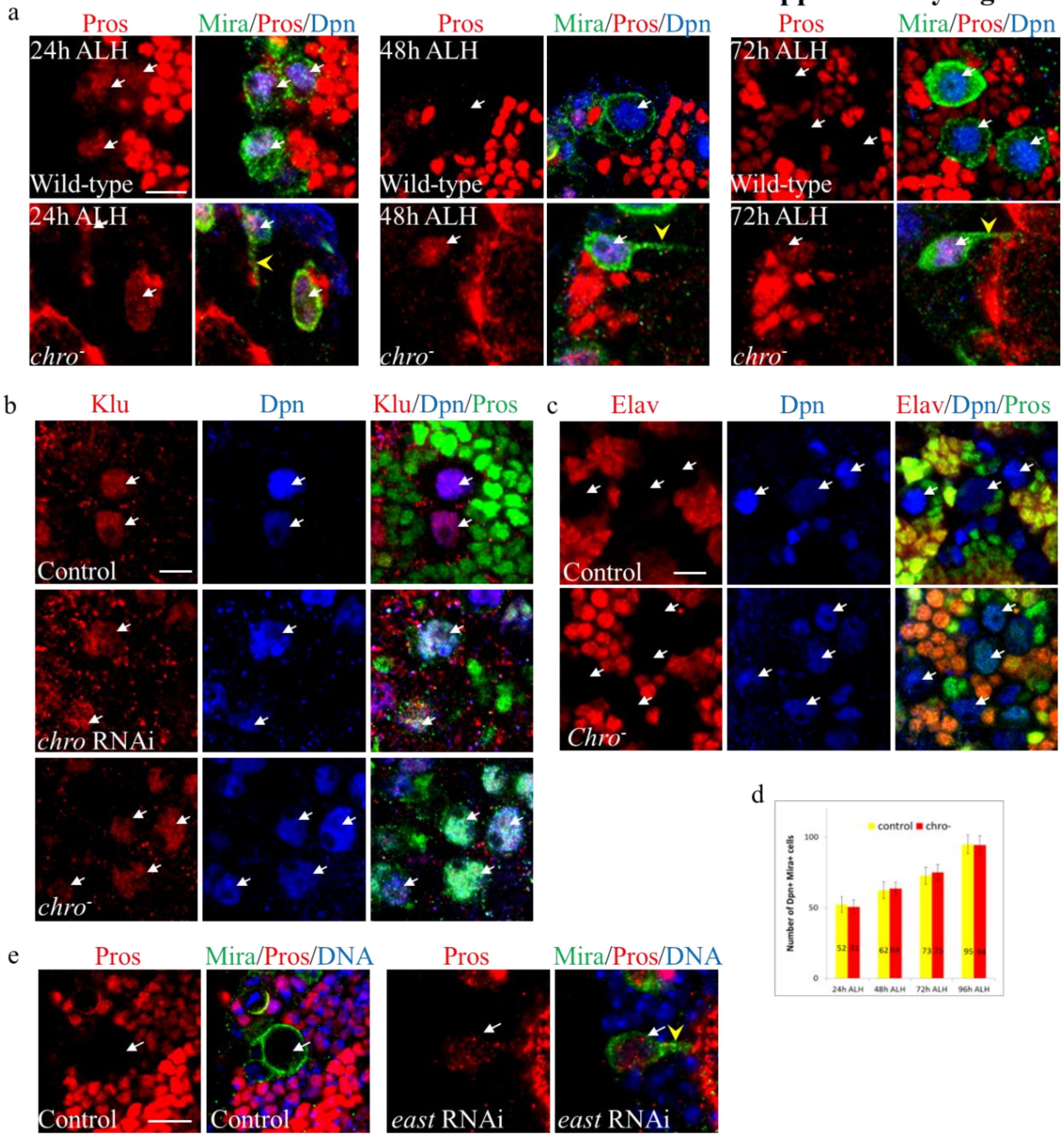

Supplementary Figure 6. Chro suppresses nuclear Pros in NSCs. (**a**) Dpn, Mira and Pros were labelled in wild-type and *chro*- mutant larval NSCs at 24h ALH, 48h ALH or 72h ALH. NSCs with nuclear Pros: wild-type, 24h ALH, 64%, n=668; *chro*-, 24h ALH, 67%, n=483; wild-type, 48h ALH, 0%, n=577; *chro*-, 48h ALH, 77%, n=482; wild-type, 72h ALH, 0%, n=601; *chro*-, 72h ALH, 80%, n=625. (**b**) NSCs in control, *chro* RNAi knockdown and *chro*- mutant were labelled with Pros, Klumpfuss (Klu) and Dpn. (**c**) Pros, Elav and Dpn were labelled in control and *chro*- mutant NSCs. (**d**) Quantification of Dpn^+ $Mira^+$ cells per central brain hemisphere in control

and *chro*$^{-}$ mutant at various time points. Control, 24h ALH, 52±6, n=21; *chro*$^{-}$, 24h ALH, 51±5, n=21; control, 48h ALH, 62±6, n=21; *chro*$^{-}$, 48h ALH, 64±5, n=20; control, 72h ALH, 73±6, n=21; *chro*$^{-}$, 72h ALH, 75±6, n=22; control, 96h ALH, 95±7, n=21; *chro*$^{-}$, 24h ALH, 94±6, n=21. Error bars indicate ± s.d.. (**e**) Mira, Pros and Topro-3 (for DNA) were labelled for the NSCs in control and *east* RNAi knockdown. White arrows indicated NSCs in a-c, e, arrowheads indicated the cellular extension of NSCs in a, e. Scale bars, 10 µm.

Supplementary Figure 7

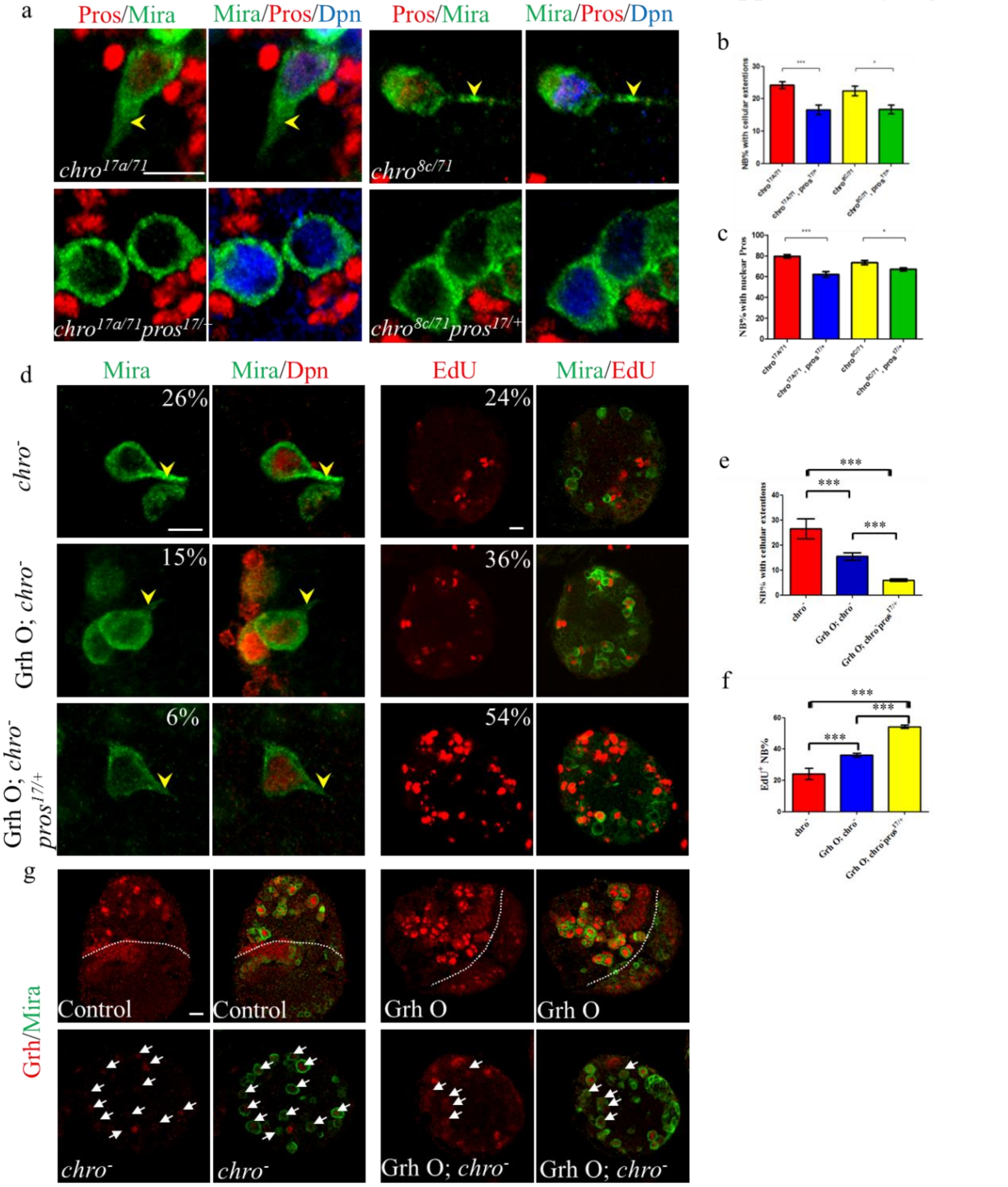

Supplementary Figure 7. Genetic interactions among *chro*, *grh* and *pros*. (**a**) Dpn, Mira and Pros were labelled in NSCs from *chro*$^{17a/71}$, *chro*$^{17a/71}$ *pros*$^{17/+}$, *chro*$^{8c/71}$ and *chro*$^{8c/71}$ *pros*$^{17/+}$. (**b-c**) Quantification of NSCs with cellular extensions or with nuclear Pros in various genotypes: NSCs with cellular extensions: *chro*$^{17a/71}$, 24%, n=739; *chro*$^{17a/71}$ *pros*$^{17/+}$, 17%, n=644; *chro*$^{8c/71}$, 22%, n=683; *chro*$^{8c/71}$ *pros*$^{17/+}$, 17%, n=590. NSCs with nuclear Pros: *chro*$^{17a/71}$, 80%, n=867; *chro*$^{17a/71}$

pros$^{17/+}$, 62%, n=688; *chro*$^{8c/71}$, 74%, n=806; *chro*$^{8c/71}$ *pros*$^{17/+}$, 67%, n=721. (**d**) Mira and Dpn or EdU and Mira were co-labelled in *chro*$^{-}$, *insc*>UAS-Grh O; *chro*$^{-}$ (Grh O; *chro*$^{-}$) and *insc*>UAS-Grh O; *chro*$^{-}$*pros*$^{17/+}$ (Grh O; *chro*$^{-}$ *pros*$^{17/+}$). (**e-f**) Quantification of NSCs with cellular extensions or with EdU incorporation in various genotypes: NSCs with cellular extensions: *chro*$^{-}$, 26%, n=755; Grh O; *chro*$^{-}$, 15%, n=1098; Grh O; *chro*$^{-}$ *pros*$^{17/+}$, 7%, n=735; NSCs with EdU incorporation: *chro*$^{-}$, 24%, n=584; Grh O; *chro*$^{-}$, 36%, n=523; Grh O; *chro*$^{-}$ *pros*$^{17/+}$, 54%, n=1336. (**g**) Grh and Mira were labelled in larval brains from control, *insc*>UAS-Grh O (Grh O), *chro*$^{-}$ and Grh O; *chro*$^{-}$. The central brain is to the left of the white dotted line and white arrows point out those NSCs with very weak Grh in Grh O; *chro*$^{-}$ in g. All error bars indicate ± s.d. in b-c and e-f. *** indicates $p<0.001$ and * indicates $p<0.05$ in b, c, e f by student's *t*-test. Arrowheads indicated the cellular extension of NSCs in a, d. Scale bars, 10 μm.

Supplementary Table 1: Primers used for qPCR and cloning

Gene/primer name	Primer sequence (5' to 3')
ChIP-*CG30158*	F: TCTTCCGTGCTGGTGATT
	R: CTAATGAATCAGAGTGAAAAC
ChIP-*CR42836*	F: GTCAGATCGCATTTTCTGTAC
	R: GACGAAAAGGACGCCATGTTA
ChIP-*CR34702*	F: TGGAACTGACAGGCAGAGCCA
	R: TGTTATAACTACTTCAGTGCCAAT
ChIP-*IM3*	F: GGCACGCCTCTTATGATGGACATT
	R: CGCCGAATCAATAAACTGTCATAT
ChIP-*Vsx1*	F: TTGACCAAATTCAACTGAATT
	R: GCGCTCAATATTCCATTGCTG
ChIP-*Vsx1* coding	F: AACAAGCTGCCGCTCGTGGCCAGG
	R: CGTGGGCGTGGTGAGCATGAT
ChIP-*Kr*	F: TTGTATCAGTCGTGATTTGGC
	R: CAACAAATTCTTCTTGTGCTCCTA
ChIP-*pdm1*	F: GCTGATGTTTAGTCCGCGCAAAGT
	R: AGCCAAGTCAAGATCTATAAAACG
ChIP-*grh*	F: GTGAGTAATTGCCCACAAAGGGCG
	R: ACTCCAGCTGCAACTGACCGCCAT
ChIP-*grh-pro*	F: AAGACTTGCAATGATGTAGAAAAG
	R: CCTATGGTTGTTGCTATTGCTGTT
ChIP-*pros* coding	F: CGGCTGCCATGTTCCAGGCGC<br>R: ATTGCTGTTGCCGCCGTTCGA
ChIP-*pros* pro1	F: ATCAGCCACAGTTTCAG<br>R: TTTGCCGACTACTGCGGGCGC
ChIP-*pros* pro2	F: AGCACACATCGACAAACCCTTC<br>R: GTATTGGTGACGTGTGACCGT
ChIP-*pros* pro3	F: TTCATTCGCAAGTTTTTCGGG<br>R: TAAGCCAAAAGGTATCCT
ChIP-*pros* pro4	F: TCACACTCCCACCCCCACT<br>R: GAATCTCAAAAGTGTGT
ChIP-*pros* pro5	F: GAACCTACGGGCTGCA<br>R: TCTCCACTTGAGCTTATTCTC
ChIP-*pros* pro6	F: TTATACAAGAAGTGTTCT<br>R: TTCGATTTCAATTGCGAGTGC

FL: full length; F: forward; R: reverse
